# Relationship between Size of the Foveal Avascular Zone and Carbohydrate Metabolic Disorders during Pregnancy

**DOI:** 10.1155/2019/3261279

**Published:** 2019-11-04

**Authors:** Masahiko Sugimoto, Yasuko Wakamatsu, Ryohei Miyata, Hisashi Matsubara, Mineo Kondo, Yuki Kamimoto, Tomoaki Ikeda

**Affiliations:** ^1^Department of Ophthalmology, Mie University Graduate School of Medicine, 2-174 Edobashi, Tsu 514-8507, Japan; ^2^Department of Obstetrics and Gynecology, Mie University Graduate School of Medicine, 2-174 Edobashi, Tsu 514-8507, Japan

## Abstract

**Aim:**

To determine whether the area of the foveal avascular zone (FAZ), as a morphological indicator of the microcirculation of the perifoveal capillary network, changes in the carbohydrate metabolism disorders during pregnancy (the gestational age of patients with gestational diabetes mellitus (GDM) and preexisting diabetes (PexD)).

**Methods:**

Ten normal individuals and 41 eyes of 41 patients, 28 with GDM and 13 with PexD, were studied. A 3 × 3 mm area of the FAZ of the superficial capillary plexus layer (SCP) and the deep capillary plexus layer (DCP) was determined by optical coherence tomography angiography (OCTA; RS-3000 Advance, NIDEK). The significance of the correlation between the size of the FAZ and the weeks of pregnancy was determined.

**Results:**

The area of the FAZ of the SCP was 0.38 ± 0.11 mm^2^ (normal eyes), 0.41 ± 0.16 mm^2^ (GDM), and 0.43 ± 0.10 mm^2^ (PexD). The area of the FAZ of the DCP was 0.78 ± 0.23 mm^2^ (normal eyes), 0.69 ± 0.16 mm^2^ (GDM), and 0.79 ± 0.25 mm^2^ (PexD). No significant difference in the FAZ sizes was observed between the groups. The average number of weeks of pregnancy was 24.1 ± 8.2 weeks in the eyes with GDM and 23.3 ± 11.4 weeks in the eyes with PexD (*P* > 0.05). Significant correlations were found between the size of the FAZ of the SCP and the number of weeks (*r* = 0.37, *P*=0.04 for GDM, and *r* = 0.49, *P*=0.04 for PexD, Spearman's rank-order correlation coefficient).

**Conclusions:**

For GDM and PexD under established glycemic control, the area of the FAZ is not affected, but vascular changes occurred at the early phase of pregnancy.

## 1. Introduction

Adverse systemic disorders that develop during pregnancy are strongly associated with changes in the microcirculation. Pregnant patients with elevated blood sugar levels are known to develop systemic complications during pregnancy [1], and this clinical state is called “carbohydrate metabolism disorders during pregnancy (CMDP).” CMDP differs from conventional diabetes mellitus (DM) and is classified as gestational diabetes mellitus (GDM) and preexisting diabetes (PexD). Because of the risk of hyperglycemia-associated complications including congenital abnormalities, fetal macrosomia of the newborn, and systemic disorders during pregnancy, strict blood sugar control is recommended for patients with CMDP [2].

PexD during pregnancy is associated with type 1 diabetes and type 2 diabetes, and it is a significantly greater maternal and fetal risk than GDM [3]. The guidelines of the American Academy of Ophthalmology recommend that the diabetic retinopathy (DR) should be assessed during pregnancy [4].

GDM is considered to be present when any signs of blood sugar intolerance are detected after the first recognition of pregnancy [5]. Because the level of blood sugar becomes normal after birth in the most of GDM cases, the Diabetic Retinopathy Preferred Practice Pattern of the American Academy of Ophthalmology states that GDM patients do not have an increased risk for DR during pregnancy (https://www.aao.org/preferred-practice-pattern/diabetic-retinopathy-ppp-updated-2017). On the contrary, GDM patients have an increased risk of developing type 2 diabetes [6, 7]. A population-based study of 9888 patients found that GDM was an independent risk factor for long-term maternal ophthalmic morbidity [8]. Thus, it is unclear whether GDM patients require special attention for DR progression.

The mechanisms underlying CMDP may differ for subjects with regular DM or who are pregnant. Pregnancy itself is believed to be a period of immense physiological or hemodynamic changes including retinal change. Although fluorescein angiography (FA) is the standard method to evaluate the retinal vasculature, it is difficult to perform FA on pregnant women because it can be harmful to the fetus (https://www.accessdata.fda.gov/drugsatfda_docs/label/2006/021980s000lbl.pdf).

There are many other methods to evaluate the retinal vasculature and CMDP during pregnancy, for example, fundus photographs [9], laser Doppler images [10, 11], and optical coherence tomography (OCT) [12, 13]. Since none of these methods constitute a direct evaluation of the vasculature, they are not comparable to conventional FA. OCT angiography (OCTA) is recently introduced to obtain 3 × 3 OCTA en face images of the retinal vascular pattern [14, 15]. One of the structures that can be assessed with OCTA is the foveal avascular zone (FAZ). Especially, OCTA can evaluate the FAZ in detail with the superficial retinal capillary plexus (SCP) and deep retinal capillary plexus (DCP) [16].

The FAZ has been validated to be a morphological indicator of the microcirculation of the perifoveal capillary network. Earlier studies have shown that capillary closure due to diabetic vascular damage is the cause of FAZ enlargement and that the area of the FAZ is correlated with the visual acuity in eyes with DR [17–19]. OCTA can also be used to determine if there has been an enlargement of the FAZ in eyes with DR [20]. However, there is no previous detailed study of the retinal vasculature by OCTA for CMDP.

The purpose of this study was to determine whether the area of the FAZ, as a morphological indicator of the microcirculation of the perifoveal capillary network, changes in CMDP.

## 2. Methods

All of the procedures used conformed to the tenets of the Declaration of Helsinki. A signed informed consent was obtained from all of the subjects after an explanation of the procedures to be used. This was a prospective, cross-sectional study that was performed at a single institution from February 12, 2016, to December 31, 2018.

The procedures used in this study were approved by the Ethics Committee of Mie University Hospital (Tsu, Mie, Japan; No. 2982) and registered at the University Hospital Medical Network (UMIN) Clinical Trials Registry (CTR). The registration title was UMIN 000021644.

### 2.1. Study Population

The normal subjects and the patients were divided into three groups. Group I consisted of volunteers who have never been diagnosed with any systemic disease and were not pregnant. GDM was diagnosed to be present when any degree of glucose intolerance was detected with the 75 g oral glucose tolerance test during pregnancy. Women with PexD were recruited from the Department of Obstetrics and Gynecology of the Mie University Hospital. The subjects were examined at the Department of Ophthalmology of the Mie University Hospital during the gestational period.

The number of weeks of pregnancy was estimated based on the early ultrasound images. Pregnant patients in Groups II and III were divided into the early stage (<25 weeks of pregnancy as the 1st and 2nd trimesters) and the late stage (≥25 weeks of pregnancy as the 3rd trimester).

### 2.2. Patient Examinations

The data obtained from the medical records included the age, body-mass index (BMI), type of DM, duration of DM, stage of DR, blood pressure, serum creatinine levels, and gestational age. The BMI was estimated from the height and body weight at the time the samples were collected. The systolic blood pressure (SBP) and diastolic blood pressure (DBP) were measured in the upper arm with a sphygmomanometer. Examinations were performed in the sitting position after rest for 5 to 10 minutes. Blood samples were collected from the GDM and PexD patients on the same day as the ocular evaluations. The fasting blood sugar (FBS) was determined by the hexokinase/glucose-6-phosphate dehydrogenase method. FBS levels lower than 120 mg/dL were considered normal. The hemoglobin A1c (HbA1c) levels were measured by column chromatography in a range of 4.9–6.0% with the National Glycohemoglobin Standardization Program. The degree of nephropathy was determined by the serum creatinine levels in a normal range of 0.48–0.79 mg/dL.

All patients underwent comprehensive ophthalmologic examinations including slit-lamp biomicroscopy and dilated fundus examinations (YH, MS, YM, and HM). The axial length (AL) was measured by optical interferometry (Fourier-domain A-scan technology with a swept source laser; OA-2000, Tomey, Tokyo, Japan). We considered axial length corrections incorporated in the calculations because they can significantly affect the FAZ [21]. All patients underwent at least 1 examination in each trimester of pregnancy.

The inclusion criteria were women aged >20 and <50 years. The exclusion criteria were the presence of severe media opacities such as severe cataract or vitreous hemorrhage. Patients with any type of chorioretinal disease including epiretinal membrane or vitreomacular traction syndrome, glaucoma, and previous ocular surgery were also excluded. Patients with any systemic disorders at the initial examination were also excluded.

### 2.3. Optical Coherence Tomography Angiography

Because the OCTA has been validated as a noninvasive method to visualize and measure the SCP and DCP of the FAZ, we used OCTA for this study [16].

Only the right eye of each patient was evaluated by OCTA (RS-3000 Advance Instrument, Nidek, Gamagori, Aichi, Japan). The OCTA device had a light source with a central wavelength of 880 nm, an acquisition speed of 53,000 A-scans/sec, and an axial resolution of 7 µm and a transverse resolution of 20 *μ*m of the tissue. Scans of 3 × 3 mm cubes were taken with each cube consisting of 256 clusters of four repeated B-scans centered on the fovea.

The bundled software (AngioScan OCT-A software, version 1.5.5) was used to create en face images of four different retinal and choroidal layers: the SCP, the DCP, the outer retina layer (avascular), and the choriocapillaris. Based on these default automated settings, the SCP extended from the top of the internal limiting membrane to 8 *μ*m below the inner plexiform layer (IPL). The DCP extended from 13 to 88 *μ*m below the IPL. The adjustments for different axial lengths were made by the embedded software.

### 2.4. Evaluations of the Foveal Avascular Zone

Quantitative measurements of the area of the FAZ of the SCP and DCP were made using the bundled software. In brief, the border around the area where no capillaries were observed within the 3 × 3 mm sectional OCTA images around the macula was traced. The enclosed area was defined as the FAZ, and the areas were calculated automatically based on the number of pixels within the outlined area. The average of the values obtained by the 2 observers (YW and MS) was used in the statistical analyses. We also determined the correlation between the area of the FAZ and the number of weeks of pregnancy for Groups II and III.

### 2.5. Statistical Analyses

All values are presented as means ± standard deviations. Unpaired *t*-tests were used to determine the significance of the differences between two groups. Nonrepeated-measures ANOVA and post hoc *t*-tests with Scheffe's test were used to determine the significance between three groups. Spearman's rank-order correlation coefficient was used to determine the significance of the correlations between the variables. The strength of the correlation, the *r* value, was classified as follows: 0.0 to 0.19, weakly or not correlated; 0.2 to 0.39, weakly or less correlated; 0.4 to 0.69, moderately correlated; 0.7 to 0.89, strongly correlated; and 0.9 to 1.0, very strongly correlated. Statistical significance was set at *P* < 0.05. The statistical evaluations were performed by Statcal 4 Statistical Program (Statcal, OMC, Saitama, Japan).

## 3. Results

### 3.1. Clinical Characteristics

The clinical characteristics of the 10 normal participants and the 41 patients are summarized in Table 1. Group I included 10 normal females, Group II included 28 women, 14 with early-stage GDM and 14 with late-stage GDM, and Group III included 13 women, 6 with early-stage PexD and 7 with late-stage PexD. Group III consisted of 2 patients with type 1 DM and 11 patients with type 2 DM.

There was no significant difference in the age among the three groups (29.6 ± 4.6 years (range 25–40 years, median 28 years) for Group I, 34.0 ± 5.4 years (range 23–42 years, median 35 years) for Group II, and 34.0 ± 5.2 years (range 27–43 years, median 33 years) for Group III, *P*=0.13, nonrepeated-measures ANOVA). There were also no significant differences in the number of weeks of pregnancy (24.1 ± 8.2 weeks for Group II and 23.3 ± 11.4 weeks for Group III, *P*=0.17, unpaired *t*-tests) and the axial length (24.1 ± 1.3 mm for Group I, 25.3 ± 1.8 mm for Group II, and 25.1 ± 2.7 mm for Group III, *P*=0.21, unpaired *t*-tests). There were no significant differences in the SBP (113.6 ± 25.9 mmHg for Group II and 129.2 ± 28.2 mmHg for Group III, *P*=0.18, unpaired *t*-tests), in the DBP (71.2 ± 13.8 mmHg for Group II and 76.7 ± 10.6 mmHg for Group III, *P*=0.11, unpaired *t*-tests), in the serum creatinine level (0.45 ± 0.09 mg/dL for Group II and 0.47 ± 0.09 mg/dL for Group III, *P*=0.44, unpaired *t*-tests), and in the BMI (24.8 ± 5.2% for Group II and 28.0 ± 5.8% for Group III, *P*=0.12, unpaired *t*-tests). However, there were significant differences in the fasting blood glycemic control (106.0 ± 23.1 mg/dL for Group II and 126.6 ± 28.2 mg/dL for Group III, *P*=0.02, unpaired *t*-tests) and HbA1c levels (5.6 ± 0.6% for Group II and 7.3 ± 1.4% for Group III, *P*=0.000035, unpaired *t*-tests). Diabetic retinopathy (DR) was not present in all of the 41 CMDP patients.

### 3.2. Imaging and Quantification of the FAZ

The areas of the FAZ of the SCP and DCP are presented in Table 2. The difference in the size of the FAZ of the SCP among the three groups was not significant (0.38 ± 0.11 mm^2^ for Group I, 0.41 ± 0.16 mm^2^ for Group II, and 0.43 ± 0.10 mm^2^ for Group III, *P*=0.5226, nonrepeated-measures ANOVA). The difference in the sizes of the FAZ of the DCP was also not significant (0.78 ± 0.23 mm^2^ for Group I, 0.69 ± 0.16 mm for Group II, and 0.79 ± 0.25 mm for Group III, *P*=0.3941, nonrepeated-measures ANOVA).

The areas of the FAZ of the SCP and DCP at early and late stages of pregnancy are presented in Table 3. The differences in the size of the FAZ of the SCP between the pregnant groups (Group II and Group III) at the early stage were also not significant (0.47 ± 0.19 mm^2^ for Group II and 0.45 ± 0.10 mm^2^ for Group III, *P*=0.50, unpaired *t*-tests), and of the DCP (0.71 ± 0.18 mm^2^ for Group II and 0.74 ± 0.23 mm^2^ for Group III, *P*=0.67, unpaired *t*-tests). In the late stage, the sizes of the FAZ of the SCP (0.36 ± 0.09 mm^2^ for Group II and 0.41 ± 0.10 mm^2^ for Group III, *P*=0.45, unpaired *t*-tests) and of the DCP (0.68 ± 0.14 mm^2^ for Group II and 0.83 ± 0.29 mm^2^ for Group III, *P*=0.30, unpaired *t*-tests) were also not significant.

### 3.3. Correlation between the Size of the FAZ and the Number of Weeks of Pregnancy

**Figure 1:**
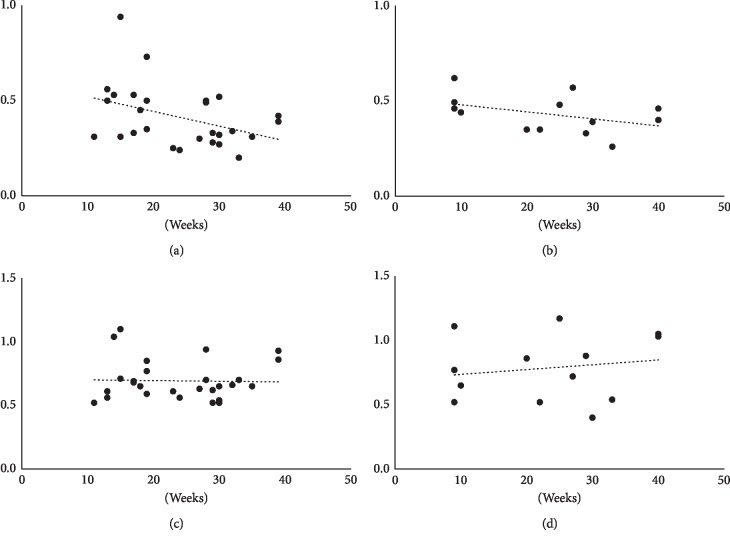
Correlation between the area of the foveal avascular zone (FAZ) and gestational age of pregnant patients with carbohydrate metabolic disorders during pregnancy. Significant moderate correlations are present between the size of the FAZ of the superficial capillary plexus (SCP) and (a) the number of weeks of pregnancy in patients with gestational diabetes mellitus (*r* = 0.37, *P*=0.04, Spearman's rank correlation coefficient) and (b) the number of weeks of pregnancy in patients with preexisting diabetes (*r* = 0.49, *P*=0.04). No significant correlation is present between the size of the FAZ of the deep capillary plexus and (c) patients with gestational diabetes mellitus (*r* = 0.01, *P*=0.47) and (d) patients with preexisting diabetes (*r* = 0.09, *P*=0.39).

There were significant moderate correlations between the size of the FAZ of the SCP and the number of weeks of pregnancy for Groups II and III (*r* = 0.37, *P*=0.04 for Group II, and *r* = 0.49, *P*=0.04 for Group III, Spearman's rank-order correlation coefficient; Figure 1). The correlation between the size of the FAZ of the DCP and the number of weeks of pregnancy was not significant in both Groups II and III (*r* = 0.01, *P*=0.47 for Group II, and *r* = 0.09, *P*=0.39 for Group III, Spearman's rank-order correlation coefficient).

## 4. Discussion

The results showed that the size of the FAZ was not significantly different in patients with CMDP from that of normal control subjects. However, there were significant correlations between the FAZ of the SCP and the number of weeks of pregnancy for CMDP.

The risk factors for a progression of DR in a patient with CMDP are similar to those for regular DR. During pregnancy, the retinal blood flow may be increased because of hyperperfusion which may then cause a progression of DR [10]. More specifically, an upregulation of circulating insulin-like growth factor-1 (IGF-1) and C-reactive peptide has been shown to be correlated with the progression of DR during pregnancy [22–25]. As a result, the retinal microvascular morphology can be altered during pregnancy [26], and its dysfunction may contribute to the diabetogenic progression during pregnancy. So, we can speculate that retinal changes occurred even in CMDP patients without DR [9–11] although the Diabetic Retinopathy Preferred Practice Pattern from the American Academy of Ophthalmology (https://www.aao.org/preferred-practice-pattern/diabetic-retinopathy-ppp-updated-2017) reported that DR is usually not present in patients with GDM.

Here, we found that the size of the FAZ of the SCP and DCP was not significantly different in both the PexD and GDM groups. Currently, technical advances have enabled clinicians to maintain good glycemic control during pregnancy, and both of our PexD and GDM groups were well controlled with an average HbA1c level lower than 8%. Thus, DR was not detected in our patients, and this may have contributed to the reduction of the risks and resulted in fewer retinal microvascular changes. So, we can conclude that glycemic control is important in reducing the diabetic retinal changes during pregnancy.

In early pregnancy, various metabolic changes occur in the womb which are associated with an organogenesis progression [27]. Such alterations are induced in part by hormones and chemical mediators secreted from the placenta which may affect the vascular conformation including that of the retina [10, 28, 29].

The sizes of the FAZ were not significantly different at early and late stages. But here we found a significant negative correlation between SCP-FAZ and the number of weeks of pregnancy. This indicates that the retinal vascular changes during pregnancy are reversible and can improve at the late stage when the vascular hemodynamics become stable.

There are some limitations in our study including the small sample size (for example, statistical power was 0.299 for a comparison between Group II and Group III) and good glycemic control for all CMDP groups.

First, a nonpregnant diabetic group and a normal pregnant group were not included in our study as control groups. We show a significant correlation with the FAZ of the SCP and the number of weeks of pregnancy, but because of the lack of the control groups, it is not clear whether this correlation is due to diabetes or pregnancy. Thus, further investigations are needed. Second, we show a significant correlation between the area of the FAZ and gestational weeks not in the DCP but in the SCP. Another group has reported a significantly larger FAZ of the DCP than the SCP in patients with DM [22]. The reproducibility of various commercialized OCT-A devices, including the RS-3000 we used, is high for normal eyes [30, 31]. However, it may affect the selection of the correct en face image to evaluate because (1) most of the commercial instruments perform layer segmentation automatically and (2) the FAZ of the DCP can be easily affected by projection artifacts [32]. And finally, the FAZ is generally considered to be anatomically a single layer of vessels where all the capillary sublayers merge. So, full-thickness slabs from OCTA images will give us detailed information. These problems will be resolved after technical improvements.

## 5. Conclusion

We have performed noninvasive retinal vascular evaluations using OCTA in CMDP patients. Our results showed that the areas of the FAZ of both the SCP and DCP were not significantly different from that of nonpregnant women controls during pregnancy under established glycemic control. This indicates the importance of glycemic control during pregnancy. We also found that the size of the FAZ is normalized as the number of weeks of pregnancy increases which indicates the possibility that some vascular changes occurred at an early phase of pregnancy with CMDP.

## Figures and Tables

**Table 1:** Clinical characteristics of each group.

	Age (years)	GA (weeks)	AL (mm)	FBS (mg/dL)	HbA1c (%)	SBP (mmHg)	DBP (mmHg)	Cr (mg/dL)	BMI (%)
Group I (normal, *n* = 10)	29.6 ± 4.6	—	24.1 ± 1.3	—	—	—	—	—	—
Group II (GDM, *n* = 28)	34.0 ± 5.4	24.1 ± 8.2	24.1 ± 1.3	106.0 ± 32.1	5.6 ± 0.6	113.6 ± 25.9	71.2 ± 13.8	0.45 ± 0.09	24.8 ± 5.2
Group III (PexD, *n* = 13)	34.0 ± 5.2	23.3 ± 11.4	25.1 ± 2.7	126.6 ± 28.2^*∗*^	7.3 ± 1.4^*∗∗*^	129.2 ± 28.2	76.7 ± 10.6	0.47 ± 0.09	28.0 ± 5.8

AL: axial length; BMI: body-mass index; DBP: diastolic blood pressure; FBS: fasting blood sugar; GA: gestational age; GDM: gestational diabetes mellitus; HbA1c: hemoglobin A1c; PexD: preexisting diabetes; SBP: systolic blood pressure. ^*∗*^*P*=0.02 and ^*∗∗*^*P*=0.000035 (unpaired *t*-test).

**Table 2:** Sizes of the FAZ of the SCP and DCP.

	FAZ of the SCP (mm^2^)	FAZ of the DCP (mm^2^)
Group I (normal, *n* = 10)	0.38 ± 0.11	0.78 ± 0.23
Group II (GDM, *n* = 28)	0.41 ± 0.16	0.69 ± 0.16
Group III (PexD, *n* = 13)	0.43 ± 0.10	0.79 ± 0.25

DCP: deep capillary plexus layer; FAZ: foveal avascular zone; GDM: gestational diabetes mellitus; PexD: preexisting diabetes; SCP: superficial capillary plexus layer.

**Table 3:** Sizes of the FAZ at early and late stages of pregnancy.

	FAZ of the SCP (mm^2^)	FAZ of the DCP (mm^2^)
Group I (normal, *n* = 10)	0.38 ± 0.11	0.78 ± 0.23
Early		
Group II (GDM, *n* = 14)	0.47 ± 0.19	0.71 ± 0.18
Group III (PexD, *n* = 6)	0.45 ± 0.10	0.74 ± 0.23
Late		
Group II (GDM, *n* = 14)	0.36 ± 0.09	0.68 ± 0.14
Group III (PexD, *n* = 7)	0.41 ± 0.10	0.83 ± 0.29

DCP: deep capillary plexus layer; FAZ: foveal avascular zone; GDM: gestational diabetes mellitus; PexD: preexisting diabetes; SCP: superficial capillary plexus layer.

## Data Availability

The data used to support the findings of this study are included within the article.
